# Differential regulation of neuronal excitability by nicotine and substance P in subdivisions of the medial habenula

**DOI:** 10.1080/19768354.2018.1456485

**Published:** 2018-03-25

**Authors:** Changwoo Lee, Soonje Lee, Changsu Woo, Shin Jung Kang, Yunhee Kim Kwon, Ki Soon Shin

**Affiliations:** a Department of Biology, Kyung Hee University, Seoul, Republic of Korea; b Department of Life and Nanopharmaceutical Sciences, Kyung Hee University, Seoul, Republic of Korea; c Department of Molecular Biology, Sejong University, Seoul, Republic of Korea

**Keywords:** Medial habenula, nicotine, substance P, addiction, withdrawal

## Abstract

The medial habenula (MHb) plays an important role in nicotine-related behaviors, such as aversion and withdrawal. The MHb is composed of distinct subregions with unique neurotransmitter expression and neuronal connectivity. Here, we showed that nicotine and substance P (SP) differentially regulate neuronal excitability in subdivisions of the MHb (ventrolateral division, MHbVL; dorsal division; MHbD and superior division: MHbS). Nicotine remarkably increased spontaneous neuronal firing in the MHbVL and MHbD, but not in the MHbS, which was consistent with different magnitudes of whole-cell inward currents evoked by nicotine in each subdivision. Meanwhile, SP enhanced neuronal excitability in the MHbVL and MHbS. Although the MHbD is composed of SP-expressing neurons, they did not respond to SP. Neurons in the MHbVL increased their firing in response to bath-applied nicotine, which was attenuated by neurokinin receptor antagonists. Furthermore, nicotine addiction and withdrawal attenuated and augmented excitatory SP effects in the MHbVL, respectively. On the whole, we suggest that MHb-involving nicotine-related behaviors might be associated with SP signaling in MHb subdivisions.

## Introduction

The MHb is an epithalamic brain region located on both sides of the third ventricle (Sutherland 1982). This region is well-preserved across all vertebrates (Concha and Wilson 2001). The MHb is a region where nAChRs are densely expressed in the brain. Previous studies have shown that nAChR subtypes such as α3, α5, and β4 are concentrated in this region, which are known to be important for nicotine-related behaviors (Picciotto et al. 1998; Perry et al. 2002; Salas et al. 2009; Fowler et al. 2011). When exposed to nicotine, neuronal excitability in these areas is greatly increased. After chronic nicotine exposure, micoroinjection of mecamylamine, an nAChR antagonist, into the MHb results in withdrawal symptoms. These results suggest that the MHb is closely related to nicotine withdrawal behavior. Among the MHb subdivisions, the ventrolateral MHb region (MHbVL) has the highest nAChR density, which is considered to play an important role in the nicotine response (Shih et al. 2014).

Although the MHb is a tiny structure, it can be divided into several subdivisions depending on neurotransmitter expression and neuronal connectivity. Each subdivision has a different density and types of nicotinic acetylcholine receptors (nAChRs) and different neuronal peptides they produce (Aizawa et al. 2012; Wagner et al. 2014). The MHb can be divided into a dorsal part and a ventral part. The ventral region of the MHb is both cholinergic and glutamatergic (Quina et al. 2009; Hsu et al. 2013). Aizawa et al. (2012) demonstrated that the dorsal part of the MHb can be further subdivided into two subdivisions according to neurotransmitter expression. Superior division of the MHb (MHbS) is exclusively glutamatergic and the dorsal region of the central part of the MHb (MHbD) is both substance P-ergic and glutamatergic.

Previous studies have suggested that the nicotine response of MHb mediates neurokinin signaling (Dao et al. 2014). They suggested that binding of nicotine to nAChR in the presynaptic terminals stimulates the release of neurokinins such as substance P (SP) and neurokinin B (NKB). The released neurokinin binds to neurokinin receptors in MHb neurons, resulting in increased neuronal excitability. Therefore, it has been speculated that nicotine-related behaviors might be related to the neurokinin responses of MHb neurons.

Although the nicotinic excitatory effect appears to be mediated by SP, neuronal responses to SP in the subdivisions of the MHb have not been examined. Therefore, we examined the neuronal effects of SP in the ventrolateral MHb (MHbVL), dorsal MHb (MHbD) and superior MHb (MHbS). Furthermore, changes in the SP responses were measured in nicotine-addiction and nicotine-withdrawal mouse models. Our study will allow us to predict the role of SP in each subdivision of the MHb in nicotine-related behaviors.

## Materials and methods

### Animals

All experiments were approved by the Kyung Hee University Animal Care and Use Committee (KHU(SE)-13-031, KHU(SE)-15-026). Mature male mice (C57BL/6, 6–8 weeks of age) were used for all experiments. The mouse were group-housed in a chamber that turned on and off the light every 12 h and 30°C temperature, 30%–60% humidity were always maintained. The food and water were always accessible.

### Nicotine addiction and withdrawal mouse model

Chronic nicotine was administered via implanted osmotic pump (Alzet, model 1004). 14–15 days before experiments, osmotic pump was implanted under the back skin of 5-week old mice to consume a fixed amount of nicotine continuously. The concentration of nicotine in osmotic pump was determined to deliver nicotine at 1 mg/kg/hr. Nicotine was dissolved in 9% NaCl. Brain slices were prepared 14 days after the osmotic pump was inserted (addicted model). In withdrawal model, 14 days after the osmotic pump implantation, mice were subjected to a second surgery to remove osmotic pumps and brain slices were prepared 24 hr after the second surgery.

### Brain slice preparation

The mice were deeply anesthetized with isoflurane and myocardial perfusion was performed with 4°C aCSF of the following composition (in mM): 124 NaCl, 2.5 KCl, 1.2 NaH_2_PO_4_, 24 NaHCO_3_, 5 HEPES, 13 Glucose, 2 MgSO_4_, 2 CaCl_2_. The brain was quickly removed after perfusion and coronally sectioned on a vibratome (VT1000s, Leica) to 250 μm submerged in 4°C aCSF. The slices were quickly transferred to a recovery solution of the following composition at 32°C (in mM): 92 NMDG, 2.5 KCl, 1.2 NaH_2_PO_4_, 30 NaHCO_3_, 20 HEPES, 25 Glucose, 5 Sodium ascorbate, 2 Thiourea, 3 Sodium pyruvate, 10 MgSO_4_, 0.5 CaCl2. After 14 min of recovery, slices were transferred to the room temperature aCSF chamber.

### Brief application of nicotine and SP

Nicotine or SP-containing glass pipet tip was positioned within 50 μm of the recorded cell's soma and the drugs were directly applied in the same direction of the bath perfusion. Nicotine (2 μM or 10 μM) and SP (50 nM or 200 nM) were puffed to the cells at a pressure of 2 psi for 100 ms using Picospritzer III (Parker). Application was controlled by Master 8 (AMPI).

### Electrophysiology

Electrophysiological recording was performed using an EPC8 amplifier (HEKA), and the signals were sampled at 10 kHz. The pipettes for Patch-clamp were pulled from borosilicate glass (Warner Instruments) and had a ∼5 MΩ tip resistance when filled with internal solution. Recovered slices were transferred to the recording chamber, was submerged with 30°C aCSF at a flow rate of 1.6 mL/min. Cells were observed through a 40x magnification video microscope (BX51WI, Olympus). The loose-seal cell-attached configuration was used for the recordings of spontaneous neuronal firing with aCSF in pipet. The currents elicited by brief nicotine or substance P treatments were recorded at −60 mV in a whole-cell mode using pipet solution contained (in mM): 100 K-Gluconate, 20 KCl, 10 HEPES, 0.2 EGTA, 10 2Na-phosphocreatine, 4 MgATP, 0.3 NaGTP; pH was adjusted to 7.2–7.3 with KOH. DATA were analyzed using Patchmaster (HEKA), Igor 6.0 (Wavemetrics Inc.) and minianalysis (synaptosoft).

## Results

### Spontaneous neuronal firings in the subdivisions of the MHb

**Figure 1.**
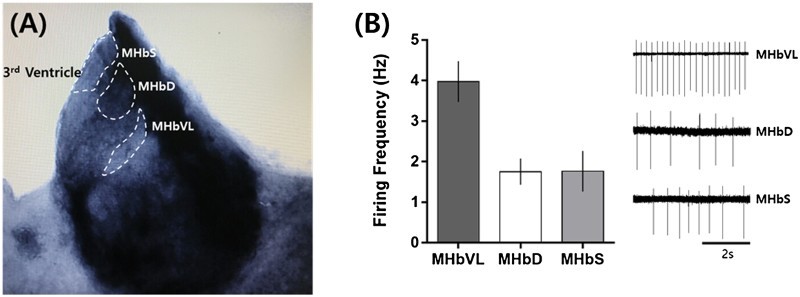
Subdivisions of the MHb and comparison of the basal firings between the subdivisions. (A) Subdivisions of the MHb investigated in this study. MHbVL: ventrolateral medial habenula, MHbD: dorsal medial habenula, MHbS: superior medial habenula. (B) Basal firing frequency and representative traces of action currents. Spontaneous firings were recorded in neurons from the subdivisions of the MHb using loose-seal cell-attached patches. MHbVL showed relatively high and very regular frequency of action potentials at 3.98 ± 0.50 Hz (*n* = 10). MHbD (1.75 ± 0.32 Hz, *n* = 15) and MHbS (1.76 ± 0.50 Hz, *n* = 7) showed low and irregular firing patterns. All values are displayed as mean ± SEM.

The locations of each subdivision in the MHb investigated in this study are shown in Figure 1(A). Recordings were performed in three subdivisions (MHbS, MHbD, and MHbVL). MHbVL neurons are both cholinergic and glutamatergic (Quina et al. 2009; Hsu et al. 2013) and highly express nicotinic acetylcholine receptors (Shih et al. 2014). MHbS neurons are exclusively glutamatergic and MHbD neurons are both substance P-ergic and glutamatergic (Aizawa et al. 2012). Using the loose-seal cell-attached patch configuration, we found spontaneous neuronal firings in three subdivisions of the MHb examined. While neurons in the MHbVL showed tonic regular firings at ∼4 Hz, neurons in the MHbD and MHbS exhibited irregular firings with much lower frequency (1 to 2 Hz) (Figure 1(B)).

### Neuronal response to nicotine in the subdivisions of the MHb

**Figure 2.**
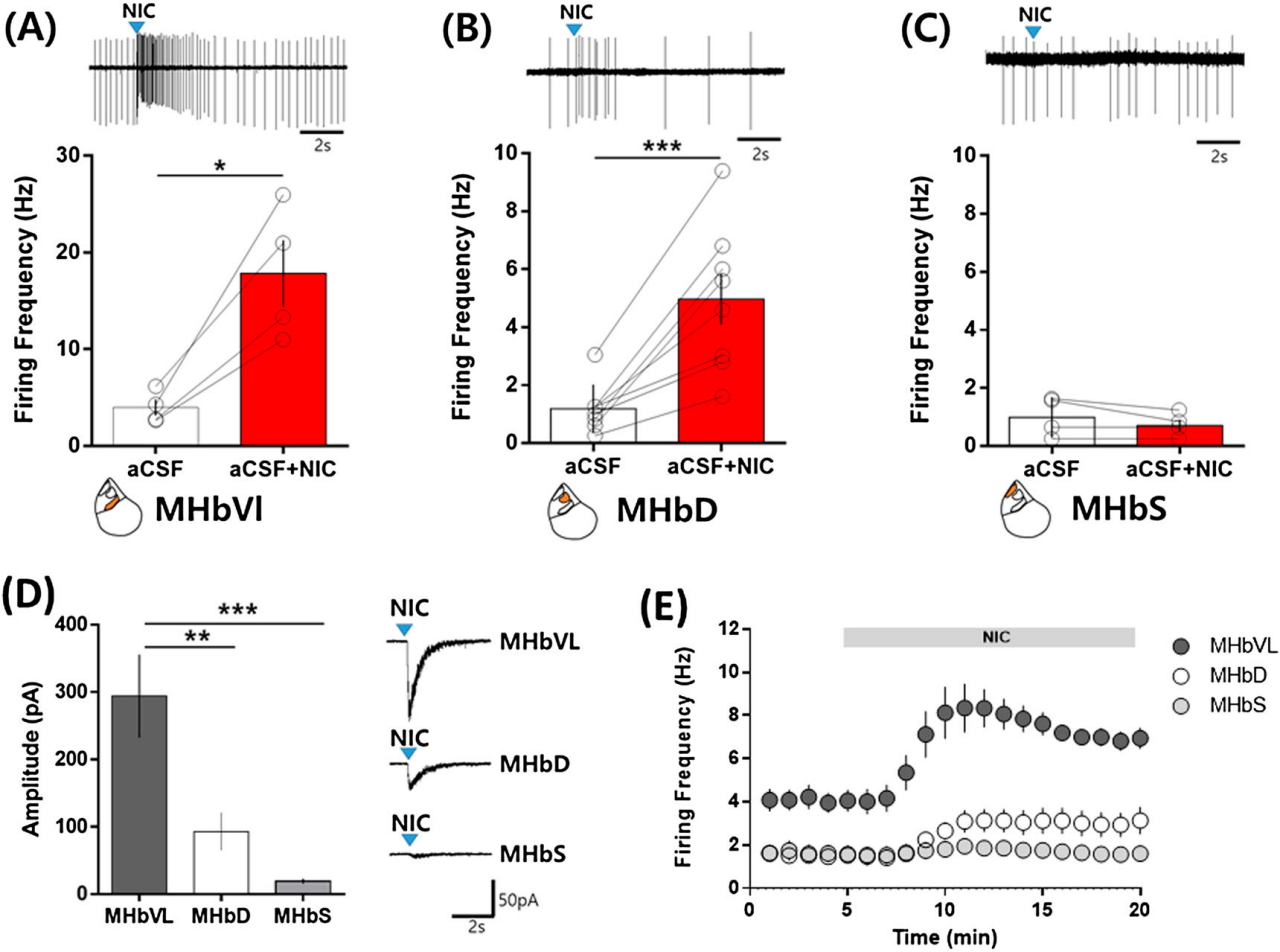
Differential effect of a brief nicotine application on neuronal excitability in the subdivisions of the MHb. (A–C) Nicotine (20 μM) was applied for 100 ms using a picospritzer and firing frequency was measured for 1-s period right after the nicotine application. (A) Neuronal firing frequency in the MHbVl was increased by nicotine treatments (Control: 3.99 ± 0.82 Hz; nicotine: 17.83 ± 3.46 Hz; *p = 0.0181, paired *t*-test, *n* = 4). (B) Neuronal firing frequency in the MHbD was increased by a brief nicotine treatment (Control: 1.19 ± 0.29 Hz; nicotine: 4.98 ± 0.89 Hz; ***p = 0.001, paired *t*-test, *n* = 8). (C) Nicotine did not alter the neuronal firing in the MHbS (Control: 0.99 ± 0.35 Hz; nicotine: 0.70 ± 0.20 Hz; *p* = 0.209, paired *t*-test, *n* = 4). (D) Nicotine-evoked whole-cell currents in subdivisions of the MHb. Nicotine (20 μM) was applied for 100 ms using a picospritzer and whole-cell currents at −60 mV were measured in the whole-cell patch configuration. Nicotine generated inward current immediately after nicotine treatment in the MHbVL (284.10 ± 61.47 pA, *n* = 6) and MHbD (92.98 ± 27.73 pA, *n* = 4). In the MHbS, nicotine-evoked currents were very small (19.28 ± 0.69 pA, *n* = 6). **P < 0.01, ***P < 0.0001, *F*_2,12_ = 15.05, one-way ANOVA followed by Newman-Keuls *post hoc* test. (E) Differential effects of continuous nicotine application on the neuronal firings in subdivisions of the MHb. Nicotine (500 nM) was continuously applied to the bath during time indicated by bar. Continuous nicotine application significantly increased neuronal firing frequency in the MHbVL (control: 3.97 ± 0.58 Hz; SP: 13.17 ± 1.35 Hz; *p* = 0.0007, paired *t*-test, *n* = 6) and in the MHbD (control: 1.53 ± 0.29 Hz; nicotine: 3.14 ± 0.62 Hz; *p* = 0.03, paired *t*-test, *n* = 9), but not in the MHbS (control: 1.48 ± 0.42 Hz; nicotine: 1.62 ± 0.30 Hz; *p* = 0.475, paired *t*-test, *n* = 7). All values are displayed as mean ± SEM.

We next examined neuronal response to a brief local puff application of nicotine in each subdivision of the MHb. 10 μM nicotine was puffed for 100 ms using a picospritzer and neuronal firing frequency was measured in the loose-seal cell-attached patch configuration. Nicotine greatly increased the basal firing of MHbVL neurons from 4 Hz to 10∼20 Hz. The increased firing was immediate and transient, and lasted for ∼3 s (Figure 2(A)). In the MHbD, nicotine also greatly increased neuronal firing from 1 Hz to 5 Hz (Figure 2(B)). In the MHbS, however, firing frequency was little changed by acute nicotine treatment (Figure 2(C)). Next, we examined whole-cell currents elicited by a brief nicotine application at −60 mV in the whole-cell patch configuration. In the MHbVL, large inward currents were observed (Figure 2(D)). Although they were smaller than the currents observed in the MHbVL, nicotine also evoked substantial currents in the MHbD (Figure 2(D)). As expected, nicotine evoked tiny currents in the MHbS (Figure 2(D)).

The long-term effect of nicotine was also examined by continuous bath application. As shown in Figure 2(E), 500 nM of nicotine was perfused from 5 min to 20 min. Nicotine increased neuronal firing frequency in the MHbVL about two-fold at the peak. The nicotine effect was slightly attenuated thereafter. In the MHbD, nicotine also increased firing frequency about 2.5-fold. There was not a significant increase in firing frequency with nicotine treatment in the MHbS (Figure 2(D)). These data were consistent with the nicotinic effects on neuronal excitability in the subdivision of the MHb by brief applications.

### Neuronal responses to SP in the subdivisions of the MHb

**Figure 3.**
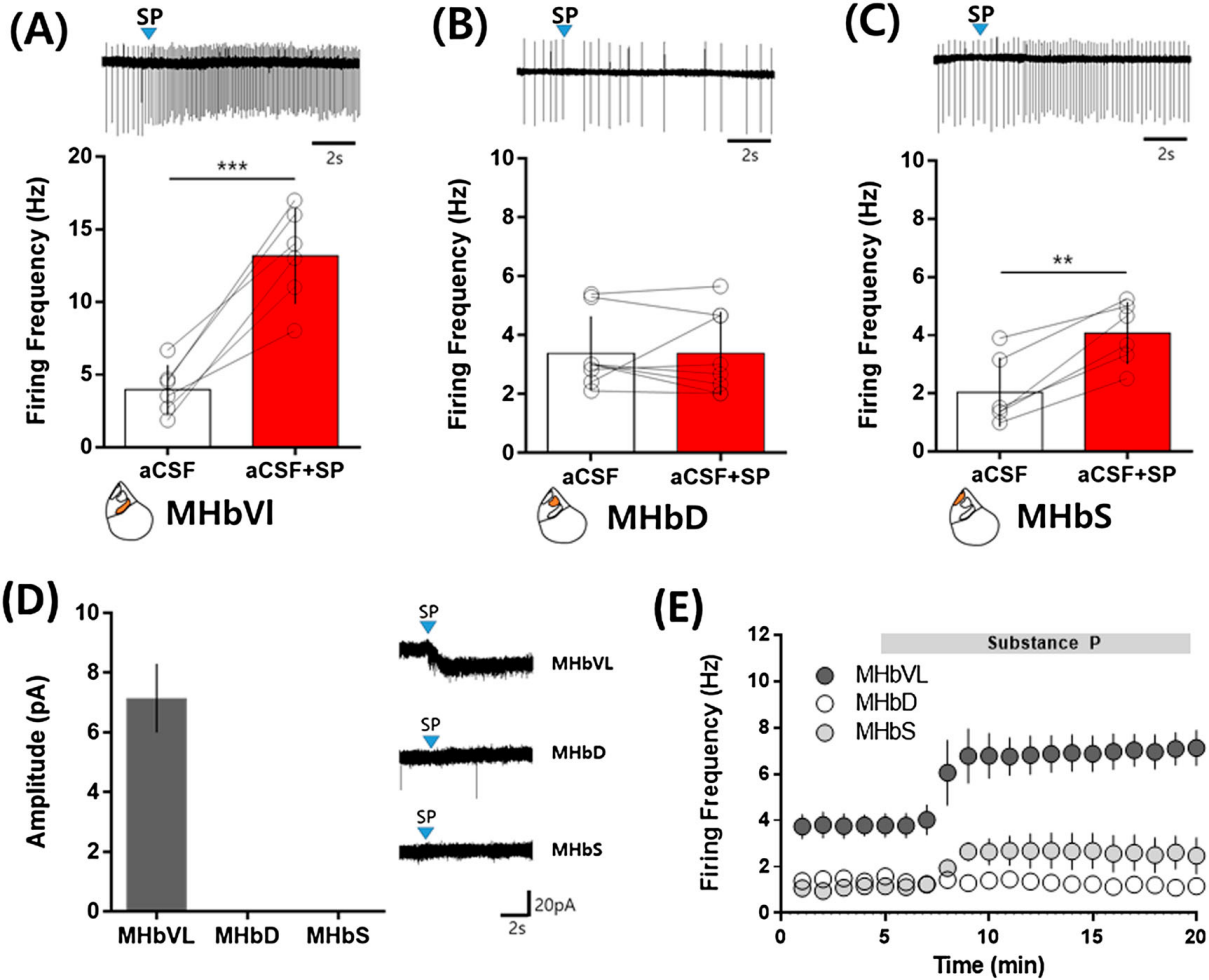
Differential effect of a brief SP application on neuronal excitability in the subdivisions of the MHb. (A–C) SP (200 nM) was applied for 100 ms using a picospritzer. Firing frequency was measured for 1-s period 2 s after SP application. (A) Neuronal firing frequency in the MHbVL was increased by nicotine treatments (Control: 3.99 ± 0.82 Hz; nicotine: 17.83 ± 3.46 Hz; *p = 0.0181, paired *t*-test, *n* = 4). (B) Neuronal firing frequency in the MHbD was changed by a brief SP treatment (Control: 3.38 ± 0.45 Hz; nicotine: 3.38 ± 0.50 Hz; *p* = 0.992, paired *t*-test, *n* = 8). (C) Neuronal firing frequency in the MHbS was increased by SP treatment (Control: 2.04 ± 0.49 Hz; SP: 4.07 ± 0.44 Hz; **p = 0.0013, paired *t*-test, *n* = 6). (D) SP-evoked whole-cell currents in subdivisions of the MHb. SP (200 nM) was applied for 100 ms using a picospritzer and whole-cell currents at −60 mV were measured in the whole-cell patch configuration. SP generated small inward current in the MHbVL (7.20 ± 1.15 pA, *n* = 5). In the MHbD (*n* = 7) and MHbS (*n* = 6), any measurable SP-evoked currents were not found. (E) Differential effects of continuous SP application on the neuronal firings in subdivisions of the MHb. SP (20 nM) was continuously applied to the bath during time indicated by bar. Continuous SP application significantly increased neuronal firing frequency in the MHbVL (control: 3.73 ± 0.52 Hz; SP: 7.08 ± 0.70 Hz; *p* = 0.0176, paired *t*-test, *n* = 3) and in the MHbS (control: 1.25 ± 0.18 Hz; nicotine: 2.65 ± 0.40 Hz; *p* = 0.0144, paired *t*-test, *n* = 8), but not in the MHbD (control: 1.28 ± 0.20 Hz; nicotine: 1.26 ± 0.28 Hz; *p* = 0.873, paired *t*-test, *n* = 4). All values are displayed as mean ± SEM.

We next examined neuronal responses to a puffed application of SP in the subdivision of the MHb. 200 nM SP was applied for 100 ms. The firing frequency of MHbVL neurons was increased about three times by SP (Figure 3(A)). The response to SP was a little delayed and long-lasting (∼1 min). Although MHbD is the only region in the MHb that expresses SP, neuronal firing in the MHbD was not affected by SP (Figure 3(B)). In the MHbS, SP increased neuronal firing about two-fold (Figure 3(C)). We also examined SP-evoked whole-cell currents in the subdivisions of the MHb (Figure 3(D)). In the MHbVL, SP evoked measurable inward currents at −60 mV. In the MHbD and MHbS, we could not observe any measurable currents evoked by SP (Figure 3(D)). Our observations on SP-evoked whole-cell currents were consistent with the SP effects on neuronal firings in the MHbVL and MHbD subdivisions neuronal firing. Considering that SP enhanced neuronal firing in the MHbS in the loose-seal cell-attached configuration, it is plausible that SP-evoked currents in MHbS neurons might not be large enough to be measured under our recording condition. It is noteworthy that SP-evoked current even in the MHbVL was only measurable right after establishing a whole-cell patch (data not shown), which implies that the SP signaling pathway requires an intact intracellular environment.

We next examined neuronal responses to continuous SP treatment in the subdivisions of the MHb. SP (20 nM) was continuously perfused from 5 min to 20 min (Figure 3(E)). Neurons in the MHbVL showed about a two-fold increase in firing frequency in the presence of SP (Figure 3(E)). In the MHbD, SP did not change neuronal firing (Figure 3(E)). In the MHbS, substance P increased firing frequency about 2.5-fold (Figure 3(E)). These data were consistent with the SP effects on neuronal excitability in the subdivision of the MHb by brief applications.

### Blockade of nicotinic excitatory effect in the presence of neurokinin receptor antagonists

**Figure 4.**
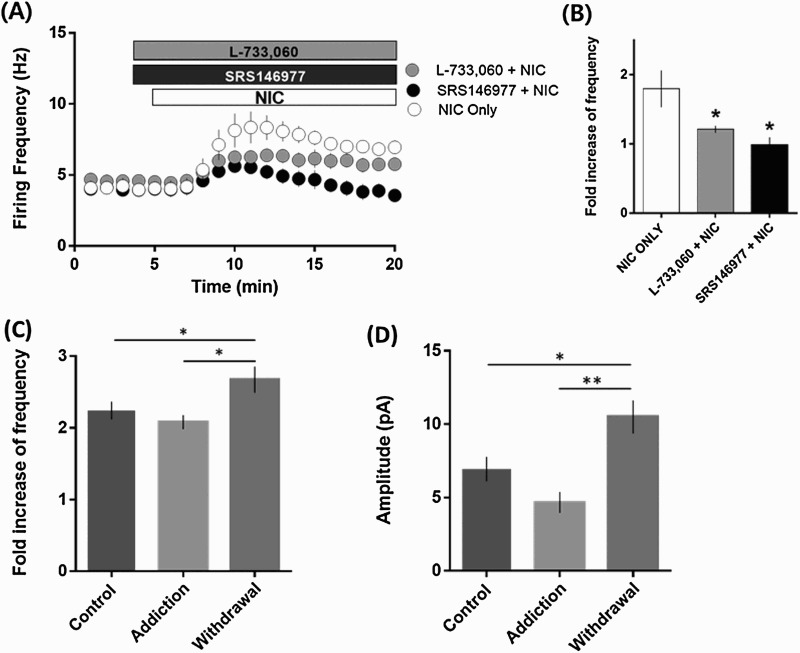
SP signaling mediated nicotinic excitatory effect in MHbVL neurons. (A and B) Blockade of nicotine effect by neurokinin receptor antagonists in the MHbVL. (A) Time-course of changes in firing frequency by nicotine in the presence of NK1 receptor antagonist (L-733,060, 10 μM, *n* = 5) and NK3 receptor antagonist (SRS146977, 10 μM, *n* = 4). Firing frequency was measured in the loose-seal cell-attached configuration. (B) Fold increase of firing frequency by nicotine in the presence of neurokinin receptor antagonists. Enhancement of firing frequency by nicotine significantly attenuated by neurokinin receptor antagonists. *P < 0.05 compared with Nic-only group, *F*_2, 9_ = 6.116, one-way ANOVA followed by Newman-Keuls *post hoc* test. (C and D) SP responsiveness of MHbVL neurons in control, addiction, and withdrawal groups. (C) Fold increase in firing frequency of MHbVL neurons from control, nicotine-addiction and withdrawal groups. SP (50 nM) was applied for 100 ms using picospritzer in the loose-seal attached configuration. SP further enhanced neuronal firings in the withdrawal group compared with control and nicotine addiction groups (control: 2.24 ± 0.12, *n* = 17; addiction: 2.08 ± 0.10, *n* = 18; withdrawal: 2.672 ± 0.18, *n* = 10; *P < 0.05 compared with control group, *F*_2, 42_ = 4.661, one-way ANOVA followed by Newman-Keuls *post hoc* test). All values are displayed as mean ± SEM. (D) SP-evoked whole-cell currents in MHbVL neurons from control, nicotine-addiction and withdrawal groups. SP (200 nM) was applied for 100 ms using a picospritzer and whole-cell currents at −60 mV were measured in the whole-cell patch configuration. nicotine withdrawal significantly increased SP-evoked currents compared to control and addiction groups (control: 6.94 ± 0.83 pA, *n* = 13; addiction: 4.66 ± 0.72 pA, *n* = 5; withdrawal: 10.49 ± 1.13 pA, *n* = 7; *P < 0.05 and **P < 0.001 compared with Nic-only group, *F*_2, 22_ = 6.784, one-way ANOVA followed by Newman-Keuls *post hoc* test). All values are displayed as mean ± SEM.

Recently, it has been reported that nicotine enhances the firings of MHb neurons via neurokinin (SP and neurokinin B) signaling using the whole-cell patch-clamp configuration (Dao et al. 2014). Since we found that SP-evoked whole cell currents require an intact intracellular environment, we considered that it would be more appropriate to examine the excitatory effect of SP in the loose-seal cell-attached configuration rather than in the whole-cell configuration. Therefore, we tested whether neurokinin receptor antagonists are involved in nicotinic excitatory effects in MHbVL neurons using the loose-seal cell-attached configuration to maintain the intracellular environment intact during electrophysiological recordings. As shown in Figure 4A, L-733,060 (10 μM), neurokinin 1 receptor (NK1: SP receptor) antagonist, and SRS146977 (10 μM), neurokinin 3 receptor (NK3: neurokinin B receptor) antagonist were pretreated 2 min before nicotine (500 nM) treatment, respectively. Both L-733,060 and SRS146977 attenuated the excitatory effect of nicotine (Figure 4(A,B)). The neurokinin receptor antagonists themselves did not change firing frequency (data not shown). These results suggest that SP signaling is involved in the long-term nicotine effect in the MHbVL, albeit partially.

### Changes of SP responses in nicotine addiction and withdrawal mouse models

Next, we examined whether the SP effect is altered in the MHbVL in nicotine addiction and nicotine withdrawal groups. Chronic nicotine was administered via an implanted osmotic pump. The osmotic pump was implanted 14 days before the electrophysiological recordings for animals to consume nicotine continuously (addiction model). In the withdrawal model, the osmotic pump was removed 14 days after implantation and recordings were made the next day. The brief application of SP (50 nM) to MHbVL neurons in the loose-seal cell-attached patch increased firing frequency in all groups. In the withdrawal group, however, SP further enhanced neuronal firings compared to the control and nicotine addiction groups (Figure 4(C)). We also examined whether nicotine addiction and withdrawal change the magnitude of SP-evoked whole-cell currents in MHbVL neurons. Although it did not reach statistical significance, SP-evoked currents (200 nM) in the addiction group were smaller than that in the control group (Figure 4(D)). Interestingly, nicotine withdrawal significantly increased SP-evoked currents compared to the control and addiction groups (Figure 4(D)). Our data showed that SP responsiveness of the MHbVL was increased in the nicotine withdrawal model, which might be related to nicotine withdrawal behaviors.

## Discussion

In this study, we examined nicotine and SP responses in the subdivisions of the MHb: MHbVL, MHbD, and MHbS. We found that both nicotine and SP remarkably increased spontaneous neuronal firing in the MHbVL. Neuronal excitability in the MHbD was increased only by nicotine, while that in the MHbS was enhanced only by SP. We also found that the nicotinic excitatory effect was associated with the neurokinin signaling pathway in the MHbVL. Furthermore, nicotine addiction and withdrawal attenuated and augmented excitatory SP effects in the MHbVL, respectively. On the whole, MHb-involving nicotine-related behaviors might be associated with SP signaling in the MHbVL.

Among the MHb subdivisions, the MHbVL has the highest nAChR density, which is considered to play an important role in the nicotine response (Shih et al. 2014). Microinjection of mecamylamine, a nicotinic acetylcholine receptor antagonist, into the MHb after chronic nicotine exposure produces nicotine withdrawal symptoms (Salas et al. 2009). Recently, it has been suggested that the nicotine response of the MHb mediates neurokinin signaling (Dao et al. 2014). According to the model from this study, SP and NKB are involved in the nicotine response in the MHb *via* activation of the neurokinin 1 and neurokinin 3 receptors, respectively: Because extensive colocalization of SP and NKB mRNAs in neurons of the MHb was found in rat (Shughrue et al. 1996), nicotine excites MHb neurons to release SP and NKB. The released neurokinin binds to neurokinin receptors in MHb neurons, resulting in increased neuronal excitability.

As expected, we found that nicotine greatly increased neuronal excitability in the MHbVL and that the nicotinic excitatory effect in this region was mediated by NK1 and NK3 receptors, albeit partially. While Dao et al. (2014) used the whole-cell patch-clamp configuration, we recorded spontaneous neuronal firings using the loose-seal cell-attached configuration. Compared with the measurement in whole-cell recording, which may be distorted due to the dialysis of intracellular components, the loose-seal cell-attached recording method is more adequate to measure spontaneous firings without perturbing the intracellular environment. In fact, we found that SP-evoked currents in whole-cell recordings were only observed right after whole-cell establishment.

It has been found that using mice chronically treated with nicotine *via* subcutaneous osmotic minipumps, microinjection of neurokinin receptor antagonists into the MHb precipitates withdrawal behavior (Dao et al. 2014). Therefore, nicotine-related behaviors might be related to the neurokinin responses of MHb neurons. Interestingly, our study showed that chronic nicotine exposure reduced SP responsiveness in MHbVL neurons, both spontaneous firings and SP-evoked whole-cell currents. On the contrary, nicotine withdrawal further potentiated SP-induced enhancement of both spontaneous neuronal firings and whole-cell currents. It has been reported that nicotine withdrawal further increases nicotine-induced enhancement of spontaneous firing frequency in the ventral MHb compared with the control (Görlich et al. 2013). Therefore, it is plausible that the nicotine-induced enhancement of neuronal excitability might be attributed to the potentiation of MHbVL responsiveness to SP during withdrawal. In conclusion, our study further supports the role of SP in nicotine-related behaviors involving MHb excitability.
